# Correlation between brain symmetry index and motor function in Parkinson’s disease: a cross-sectional study

**DOI:** 10.3389/fnins.2026.1777368

**Published:** 2026-02-24

**Authors:** Xianling Xu, Jinfeng Xu, Yuqing Zhao, Jian Song, Haoping Gu, Wei Wei, Haoran Shi, Xiehua Xue

**Affiliations:** 1 Rehabilitation Hospital Affiliated to Fujian University of Traditional Chinese Medicine, Fuzhou, Fujian, China; 2 Fujian Provincial Key Laboratory of Cognitive Function Rehabilitation, Fuzhou, Fujian, China; 3 Fujian Provincial Key Laboratory of Rehabilitation Technology, Fuzhou, Fujian, China

**Keywords:** electroencephalography, gait impairment, motor function, pairwise derived brain symmetry index, Parkinson’s disease

## Abstract

**Introduction:**

Parkinson’s disease (PD) present with asymmetric motor impairments in the different stages. The study aims to explore the pair-wise derived brain symmetry index (pdBSI) of electroencephalography (EEG) for the stage in PD.

**Materials and methods:**

96 early Stage (ePD), 85 advanced Stage (aPD) and 67 healthy individuals were recruited. EEG and MDS-UPDRS Scale were applied to the study. pdBSI of EEG was calculated and divided into the frontal, the central and the posterior regions.

**Results:**

The PD Group exhibited significant differences in pdBSI across the full-frequency band in the whole-brain, especially in the central region. The PD groups showed higher levels of *α*-pdBSI, β1-pdBSI, and β2-pdBSI in the whole-brain than the healthy group. Furthermore, the ePD group showed higher levels of *θ*-pdBSI, *α*-pdBSI, β1-pdBSI and β2-pdBSI in the central region than the healthy group. The same tendency was observed between the aPD and healthy group. The β2-pdBSI of the posterior regions in ePD and aPD was higher than the healthy group. The results showed that ePD had the highest pdBSI level, followed by the aPD and healthy group. There was a positive correlation between β2-pdBSI of the central and posterior regions with the gait sub-score of the UPDRS-III in the aPD group.

**Conclusion:**

There were hemispheric asymmetry in the PD patients. The pdBSI was a potential electrophysiological biomarker for hemispheric asymmetry in the PD. β2-pdBSI in the central and posterior region was significantly associated with the severity of gait impairment in aPD patients. In addition, the stage-dependent gradient elucidates the dynamic shift from unilateral to bilateral network involvement. Our findings underscore that quantifying interhemispheric asymmetry, beyond examining power in isolated regions, provides unique insights into the pathophysiology and clinical progression of PD.

## 1 Introduction

Parkinson’s disease (PD) is the second most common progressive neurodegenerative disorder (Warnecke et al., 2023). It is characterized by degeneration of the nigrostriatal dopaminergic pathway, which broadly impairs the function of the basal ganglia–thalamocortical neural network. This dysfunction notably involves altered physiology/activity in the primary motor cortex (M1) and supplementary motor area (SMA), leading to aberrant neural activity within motor circuits and resulting in classic motor symptoms including bradykinesia, resting tremor, rigidity, and postural instability (Burciu and Vaillancourt, 2018; Leodori et al., 2022).

PD patients often present with asymmetric motor impairments in the early stages, which typically begin unilaterally and gradually progress to bilateral involvement as the disease advances. This lateralized pattern of motor dysfunction originates from the asymmetric degenerative changes in the basal ganglia (BG) and corresponding unilateral dopamine depletion (Riederer et al., 2018; Riederer and Sian-Hülsmann, 2012). While neuroimaging techniques such as fMRI and PET provide valuable structural and functional insights, they are often resource-intensive and may not capture neural dynamics with high temporal resolution. Therefore, it is an urgent need to develop objective, accurate, and cost-effective biological markers for clinical use.

Electroencephalography (EEG) is a non-invasive auxiliary examination method that assesses brain activity with high temporal resolution, capable of capturing rapid dynamic changes in neural processes (Waninger et al., 2020). EEG is well-suited to measuring oscillatory activity due to its high temporal resolution. Abnormal beta band (15–30 Hz) synchronization has been consistently correlated with the severity of motor deficits in PD (Gatev et al., 2006; Witcher et al., 2014; Nwogo et al., 2022; Wang and Choi, 2020). The pdBSI is a set of quantitative parameters derived from EEG power spectra that objectively reflects functional asymmetry between the cerebral hemispheres by measuring power differences across frequency bands (Wang et al., 2023). This index has been validated in contexts such as post-stroke motor recovery, which reduced pdBSI in low-frequency bands over motor regions correlates with better functional outcomes (Mauro et al., 2024; Agius et al., 2017; Gao et al., 2025). However, existing EEG studies in PD have largely focused on spectral power or network connectivity, and the application of quantitative symmetry indices such as the Brain Symmetry Index (BSI) or its pdBSI in this population remains limited, and its application in evaluating PD-related motor dysfunction remains unverified.

This study innovatively introduces pdBSI to assess motor staging in PD, aiming to characterize its disease-stage-dependent variations and correlation with motor impairment severity. Given that this study aims to characterize interhemispheric asymmetry in PD patients during a stable, routinely medicated state, all clinical and EEG assessments were conducted during the unified “ON” medication period. This ensures maximal comparability across participants and focuses the analysis on pathophysiological associations during the medication efficacy phase. This approach thereby provides new electrophysiological evidence for constructing an objective, EEG-based system for dynamic monitoring and staging of PD.

## 2 Materials and methods

### 2.1 Participant characteristics

**Figure 1:**
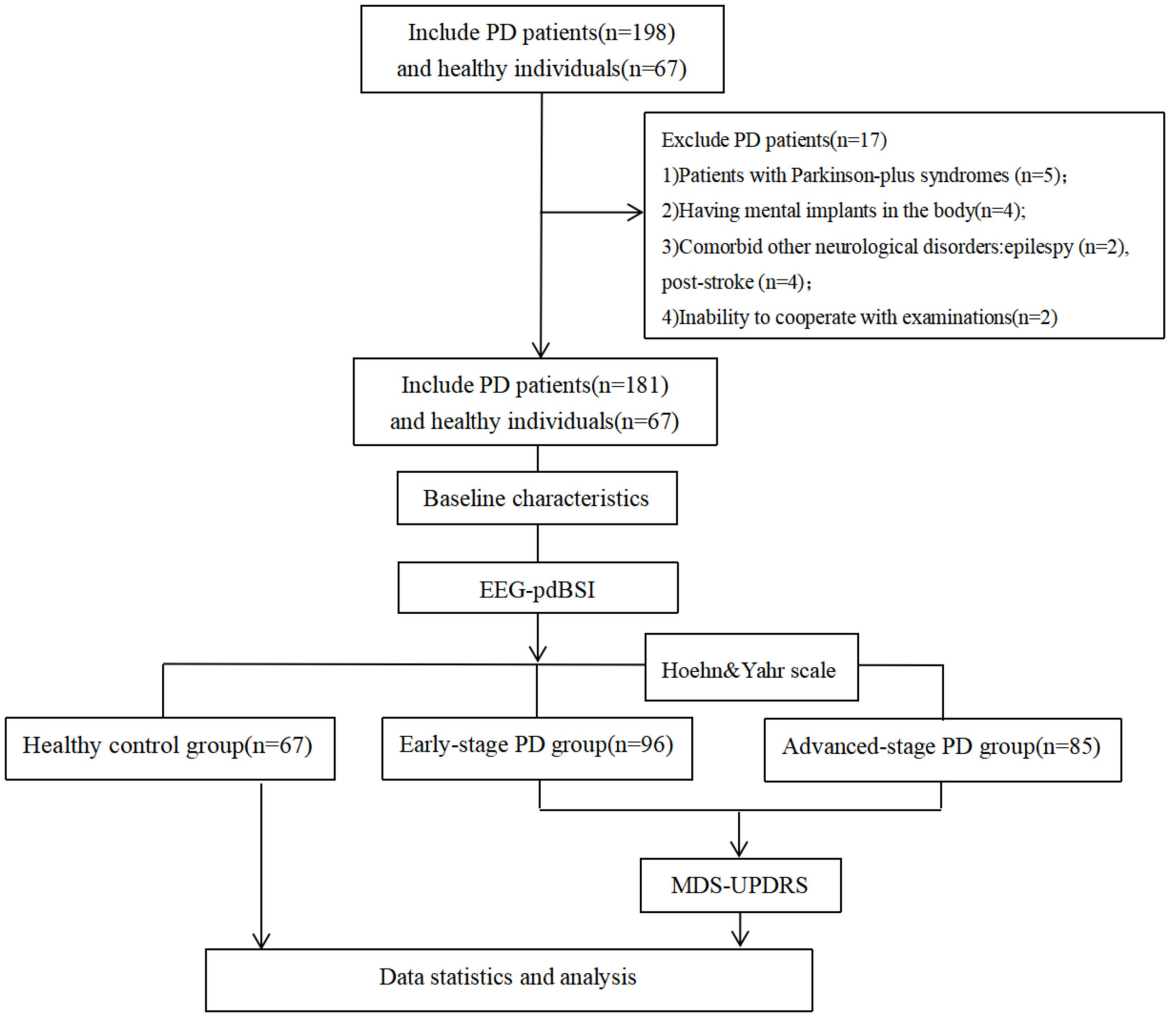
Flowchart.

This study enrolled 181 Parkinson’s patients treated at the Rehabilitation Hospital Affiliated to Fujian University of Traditional Chinese Medicine between March 2024 and June 2025. Stage 1–2 as Early Stage, Stage 2.5–5 as Advanced Stage according to Hoehn and Yahr (H–Y) classification. 96 early-stage patients and 85 advanced-stage patients were ultimately included. 67 age- and gender-matched healthy individuals were concurrently recruited as the control group (Figure 1).

Inclusion criteria: (1) Meeting diagnostic standards for ‘Parkinson’s disease’ per the international expert *MDS Clinical Diagnostic Criteria for Parkinson’s Disease (2015)* (Postuma et al., 2015); (2) Aged between 45 and 80 years; (3) Absence of severe complications or comorbidities requiring no special treatment; (4) Receiving anti-PD medication for at least 1 month prior to enrollment; (5) Voluntary participation with signed informed consent.

Exclusion criteria: (1) Patients with secondary Parkinsonism or Parkinson-plus syndromes; (2) Having mental implants in the body; (3) Comorbid other neurological disorders; (4) Patients with psychiatric disorders, severe cognitive/hearing/visual impairments, or inability to cooperate with examinations; (5) Current use of neuroleptic drugs; (6) Participation in other clinical trials that may interfere with this study’s results.

This study was approved by the Ethics Committee of Rehabilitation Hospital Affiliated to Fujian University of Traditional Chinese Medicine (Ethics Approval No.: 2025YJS-024-02). All participants provided written informed consent prior to the trial.

### 2.2 Clinical functional assessment

#### 2.2.1 Modified Hoehn and Yahr (H–Y) staging scale

The Modified Hoehn and Yahr Staging Scale comprises 7 stages: Stage 1 manifests as unilateral limb dyskinesia; Stage 1.5 shows unilateral symptoms with trunk involvement; Stage 2 exhibits bilateral motor symptoms; Stage 2.5 features mild postural reflex impairment (demonstrating postural instability during the “pull test” that can be self-corrected) in addition to bilateral symptoms; Stage 3 presents with significant bilateral symptoms and uncorrectable postural imbalance; Stage 4 displays severe functional impairment with substantial limitations in self-care abilities; Stage 5 manifests as inability to walk and complete loss of self-care ability, requiring full dependence on a wheelchair or being bedridden.

#### 2.2.2 Movement disorder society-unified Parkinson’s disease rating scale (MDS-UPDRS)

Comprising four subscales: UPDRS-I assesses cognition, psychiatric symptoms, behavior, and pain through 13 items (total score: 52 points); UPDRS-II evaluates activities of daily living through 13 items (total score: 52 points); UPDRS-III measures motor examination via 18 items (total score: 132 points). The following motor sub-scores were derived from UPDRS-III items: Rigidity: item 3.3 (neck, right/left upper/lower extremity), Bradykinesia: Items 3.4–3.8 (Finger Tapping, Hand Movements, Pronation-Supination Movements of Hands, Toe Tapping, Leg Agility) and Item 3.14 (Body Bradykinesia/Hypokinesia), Tremor: Items 3.15–3.18 (Postural Tremor of Hands, Kinetic Tremor of Hands, Resting Tremor Amplitude and Constancy), Gait: items 3.9–3.13 (freezing, postural stability); UPDRS-IV assesses motor complications, including dyskinesia, motor fluctuations, and dystonia, comprising 6 items with a total score of 24 points. This scale comprehensively evaluates the severity of motor function, non-motor symptoms, and motor complications through cumulative scoring of each assessment item, demonstrating reliability and high sensitivity. Higher scores indicate worse functionality in Parkinson’s patients. Before conducting the UPDRS assessments, detailed medical history, medication records, and the time of last medication administration were documented for each participant. If significant motor fluctuations exist, determine whether the patient is in “OFF” or “ON” periods“. Patients were classified into five severity levels based on symptom presentation: 0: Normal, 1: Minimal, 2: Mild, 3: Moderate, 4: Severe. All scores were recorded as integers, with a maximum total of 260 points. Higher scores indicate more severe symptoms. In line with the EEG data acquisition, the scale assessments were conducted following a standardized protocol: all were performed in the morning, 60–90 minutes after medication intake, and while patients were in the confirmed “ON” state, ensuring clinical–electrophysiological alignment by two trained neurologists (inter-rater reliability ICC > 0.85).

### 2.3 EEG data acquisition and processing

To ensure this alignment, the EEG recordings were also performed under standardized conditions. All EEG recordings were performed in the morning while PD patients were in the confirmed “ON” medication state. Medication timing and dose were standardized: recordings were conducted 60–90 min after the last levodopa equivalent dose (LEDD). This paradigm was chosen to prioritize clinical applicability and patient-centered research: (1) it examines brain function during patients’typical functional state, increasing the ecological validity of findings; (2) it ensures patient safety and comfort, which is paramount for reliable data collection; and (3) it allows for the identification of state-dependent biomarkers relevant to treated Parkinsonism. To mitigate confounding from medication timing and dose, a strict standardization protocol was followed.

Subjects remained awake with eyes closed in a quiet, light-shielded environment. Resting-state EEG data were acquired using the NVX52 EEG Acquisition System (Nanjing NeuroMed Technology Group Co., Ltd., China) with 19 AgCl electrodes secured via an adjustable cap. Electrode AA (We use two electrodes, A1 and A2. AA = (A1 + A2) /2) served as the reference, with impedance maintained below 20 kΩ (Lee et al., 2013). Resting-state EEG was recorded for 3 min per subject. Preprocessing of collected EEG data was performed using the EEGLAB toolbox in MATLAB (R2017b). We applied 0.5–45 Hz band-pass filtering at a sampling rate of 500 Hz. Artifactual components related to ocular movements, cardiac activity, and muscle noise were removed using Independent Component Analysis (ICA) implemented in EEGLAB. Component identification was performed through a two-step procedure: (1) automatic classification using the ICLabel plugin (version 1.5); (2) subsequent visual inspection by an experienced researcher. Components with a probability greater than 90% of being “Eye” or “Muscle” activity according to ICLabel, or those exhibiting characteristic topographic and temporal patterns of artifacts upon visual inspection, were rejected. On average, 1–3 components were removed per subject. To mitigate the impact of low-frequency drifts and sweat artifacts on theta/alpha band analyses, a 0.5 Hz high-pass filter was applied during preprocessing (in addition to the 0.5–45 Hz band-pass filter), and components with a low-frequency spectral profile were scrutinized during the visual inspection stage. A 50 Hz notch filter was additionally used to suppress line noise.

**Figure 2:**
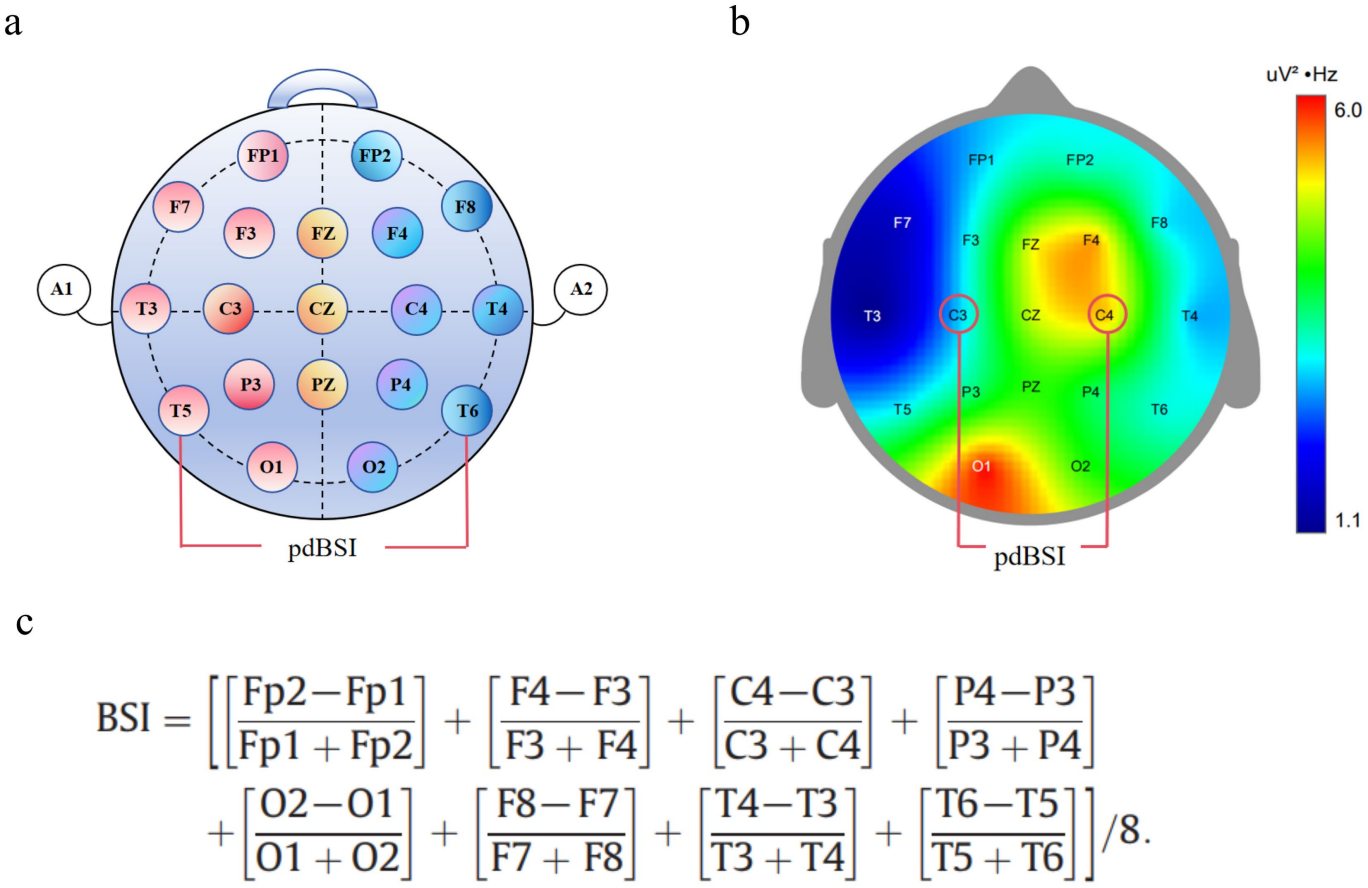
Schematic diagram of pdBSI. **(a)** Schematic diagram of the EEG cap electrodes. **(b)** EEG topographic map. **(c)** Calculation formula of pdBSI.

For preprocessed EEG data, Welch’s periodogram method calculated the Power Spectral Density (PSD) for each channel per trial. The pdBSI was computed by averaging the PSD across homologous left–right hemisphere electrode pairs. This process was repeated within the 0.5–4 Hz (Delta, *δ*), 4–8 Hz (Theta, *θ*), 8–13 Hz (Alpha, *α*), 13–20 Hz (Beta1, β1), and 20–30 Hz (Beta2, β2) frequency bands to derive band-specific pdBSI values. Regional pdBSI was calculated for: Global (F3, F4, F7, F8, C3, C4, T3, T4, P3, P4, T5, T6, O1, O2), Frontal (F7, F3, F4, F8), Central (T3, C3, C4, T4), and Posterior (T5, P3, P4, T6) regions. This index quantifies asymmetry between left–right hemispheric mirror regions by computing the mean absolute power difference across the 1–25 Hz frequency range, ranging from 0 (perfect symmetry) to 1 (maximum asymmetry). The specific algorithm is as follows (Schleiger et al., 2014) (Figure 2).

### 2.4 Statistical analysis

Statistical analysis was performed using SPSS version 27.0. Prior to group comparisons, inter-rater reliability for the UPDRS-III assessment was evaluated in a subset of 15 patients, yielding excellent agreement between two neurologists (ICC > 0.85). Continuous variables conforming to normal distribution were analyzed using one-way ANOVA; non-normally distributed data underwent the Kruskal–Wallis test, with Bonferroni correction applied to adjust significance values for multiple comparisons. Categorical variables were analyzed using *x*^2^ or Fisher’s exact tests. Statistically significant EEG parameters were correlated with clinical measures. Bivariate normally distributed data used Pearson correlation; Non-normally distributed data used Spearman’s rank correlation, reporting *r* and *p* values. FDR correction was conducted for multiple correlated results, and reporting *q* values.

To address the potential confounding effect of dopaminergic medication, the Levodopa Equivalent Daily Dose (LEDD) was included in supplementary analyses. First, the difference in LEDD between the ePD and aPD groups was compared using the Mann–Whitney *U* test. Second, to assess whether LEDD was independently associated with pdBSI measures beyond disease stage, partial correlation analyses were conducted within the combined PD cohort, controlling for disease stage (ePD vs. aPD).

## 3 Results

### 3.1 General information

There were no statistically significant differences in age or sex among the three groups. ePD and aPD groups showed no significant differences in rigidity and tremor sub-scores (*p* > 0.05) or in LEDD (*p* = 0.054), but exhibited significant differences in the MDS-UPDRS scores, bradykinesia and axial symptoms (gait/posture) sub-scores (*p* < 0.05). Normally distributed data are presented as mean ± standard deviation (*x* ± *s*), while non-normally distributed data are presented as median (25th, 75th percentiles) [*M* (25, 75%)] (Table 1).

**Table 1:** Comparison of baseline characteristics.

Indicator	HC group (*n* = 67)	ePD group (*n* = 96)	aPD group (*n* = 85)	*x*^2^/*Z*	*P*
Gender (male/female)	40/27	60/36	51/34	0.173^a^	0.917
Age (years)	69.00 (63.50, 73.00)	67.00 (63.00, 71.00)	70.00 (65.00, 72.00)	5.286^b^	0.071
MDS-UPDRS-I (score)	–	6.00 (3.00, 10.00)	9.00 (5.00, 14.00)	−3.319^c^	<0.001^*^
MDS-UPDRS-II (score)	–	9.00 (5.00, 11.00)	13.0 (9.00, 18.00)	−5.430^c^	<0.001^*^
MDS-UPDRS-III (score)	–	27.50 (19.00, 36.00)	37.50 (28.00, 46.00)	−4.645^c^	<0.001^*^
MDS-UPDRS-total (score)	–	43.00 (34.00, 56.00)	60.50 (47.00, 77.00)	−5.442^c^	<0.001^*^
Rigidity (score)	–	6.00 (2.00, 8.00)	6.00 (1.00, 8.00)	−0.144^c^	0.885
Bradykinesia (score)	–	12.00 (7.00, 16.00)	14.50 (10.00, 22.00)	−3.160^c^	0.002^*^
Tremor (score)	–	5.00 (2.00, 8.00)	4.00 (2.00, 8.00)	−0.675^c^	0.500
Gait (score)	–	3.00 (2.00, 4.00)	7.00 (5.00, 9.00)	−9.494^c^	<0.001^*^
LEDD	–	600.0 (400.0, 750.0)	650.0 (466.0, 867.5)	−1.923^c^	0.054

Data are presented as median (P25, P75), ePD: patients with Parkinson’s disease at the early stage (1–2 stage of Hoehn and Yahr); aPD: patients with Parkinson’s disease at the advanced stage (2.5–5 stage of Hoehn and Yahr); HC: health controls; pdBSI: pair wise derived brain symmetry index. MDS-UPDRS, the Movement Disorder Society-Sponsored Revision of the Unified Parkinson’s Disease Rating Scale; MDS-UPDRS I-III, the part I-III of MDS-UPDRS; Rigidity, Bradykinesia, Tremor, Gait: the subitems of UPDRS III, rigidity (item 3.3), bradykinesia (items 3.4–3.8, 3.14), tremor (3.15–3.18), and gait (3.9–3.13).

^a^*x*^2^ test.

^b^Kruskal–Wallis test.

^c^Mann–Whitney *U* test.

^*^P < 0.05.

### 3.2 Intergroup comparison of pdBSI

**Figure 3:**
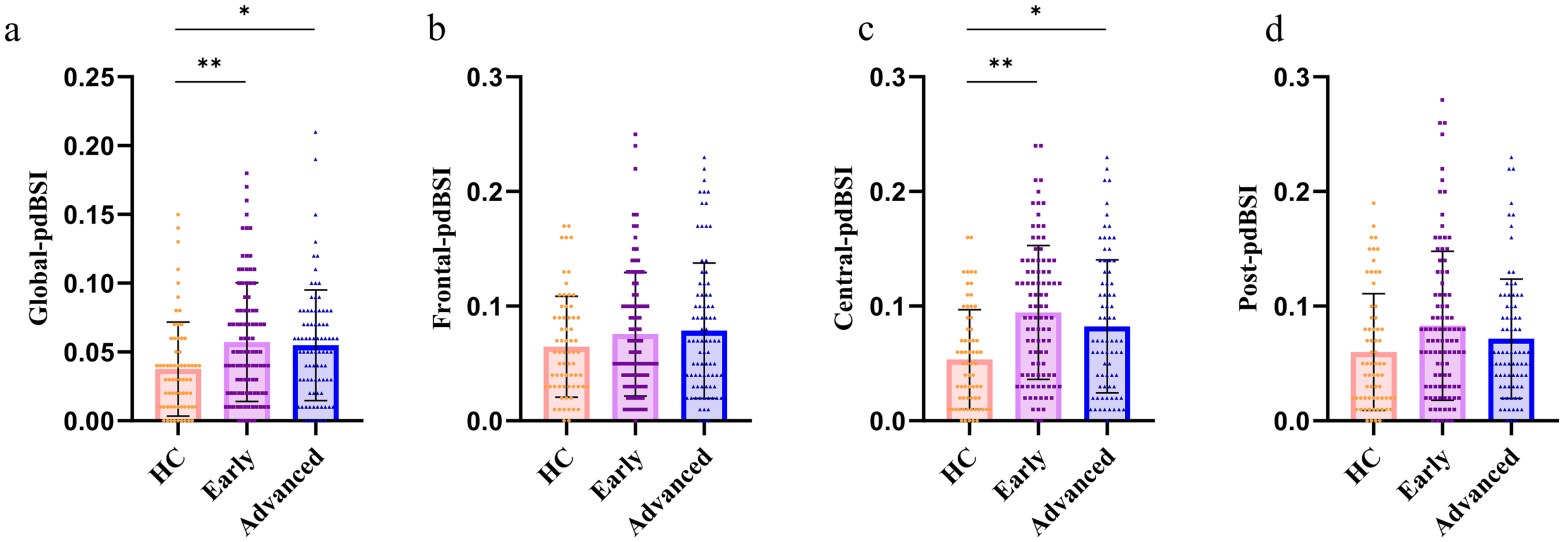
The pdBSI of the full-frequency band in the different brain regions. **(a)** The pdBSI of global region. **(b)** The pdBSI of frontal region. **(c)** The pdBSI of central region. **(d)** The pdBSI of posterior region (^*^p < 0.05, ^**^p < 0.005).

The PD group exhibited significant differences in pdBSI across the full-frequency band in the whole-brain (Figures 3a, *p* < 0.05), especially in the central region (Figure 3c, *p* < 0.001) compared with the HC group. It indicated that there was a strong association between the pdBSI of the central region and the stages of PD progression. There was no statistical significance in pdBSI in the frontal and posterior regions among the three groups (Figures 3c and 3d).

**Figure 4:**
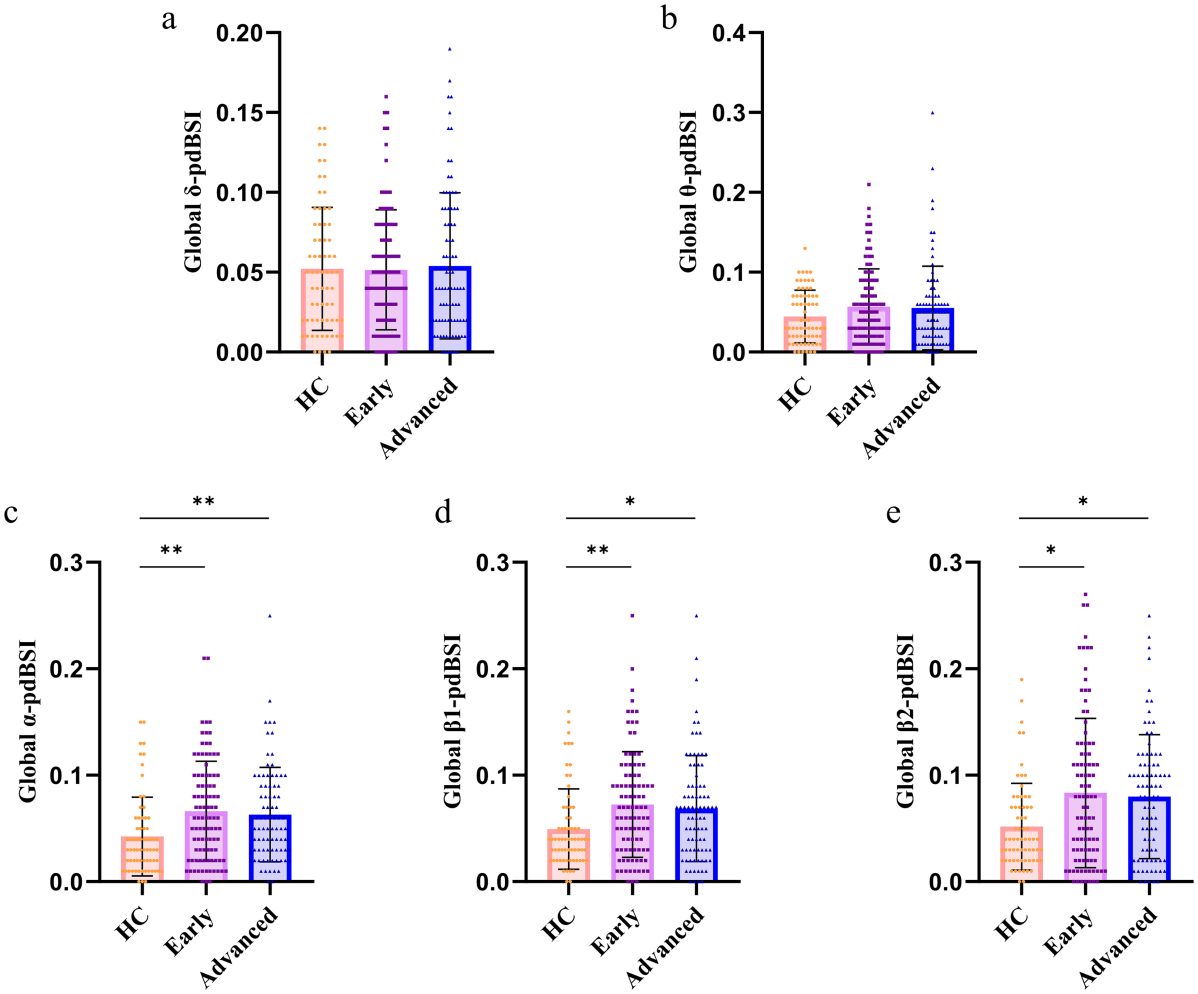
Differences in pdBSI across frequency bands in global-brain regions: **(a)** global *δ* frequency band, **(b)** global *θ* frequency band, **(c)** global *α* frequency band, **(d)** global β1 frequency band, **(e)** global β2 frequency band (^*^p < 0.05, ^**^p < 0.005).

EEG signals encompass multiple frequency bands (*δ*, *θ*, *α*, β1, β2). We analyzed changes in interhemispheric symmetry across these bands in the different stages of PD. Our findings showed that the PD groups had higher levels of *α*-pdBSI (Figure 4c, *P* < 0.005), β1-pdBSI (Figure 4d, *P* < 0.05), and β2-pdBSI (Figure 4e, *P* < 0.05) in the whole-brain regions than the HC group. No between-group differences were found in the δ and θ bands in the whole-brain regions (Figures 4a and 4b).

**Figure 5:**
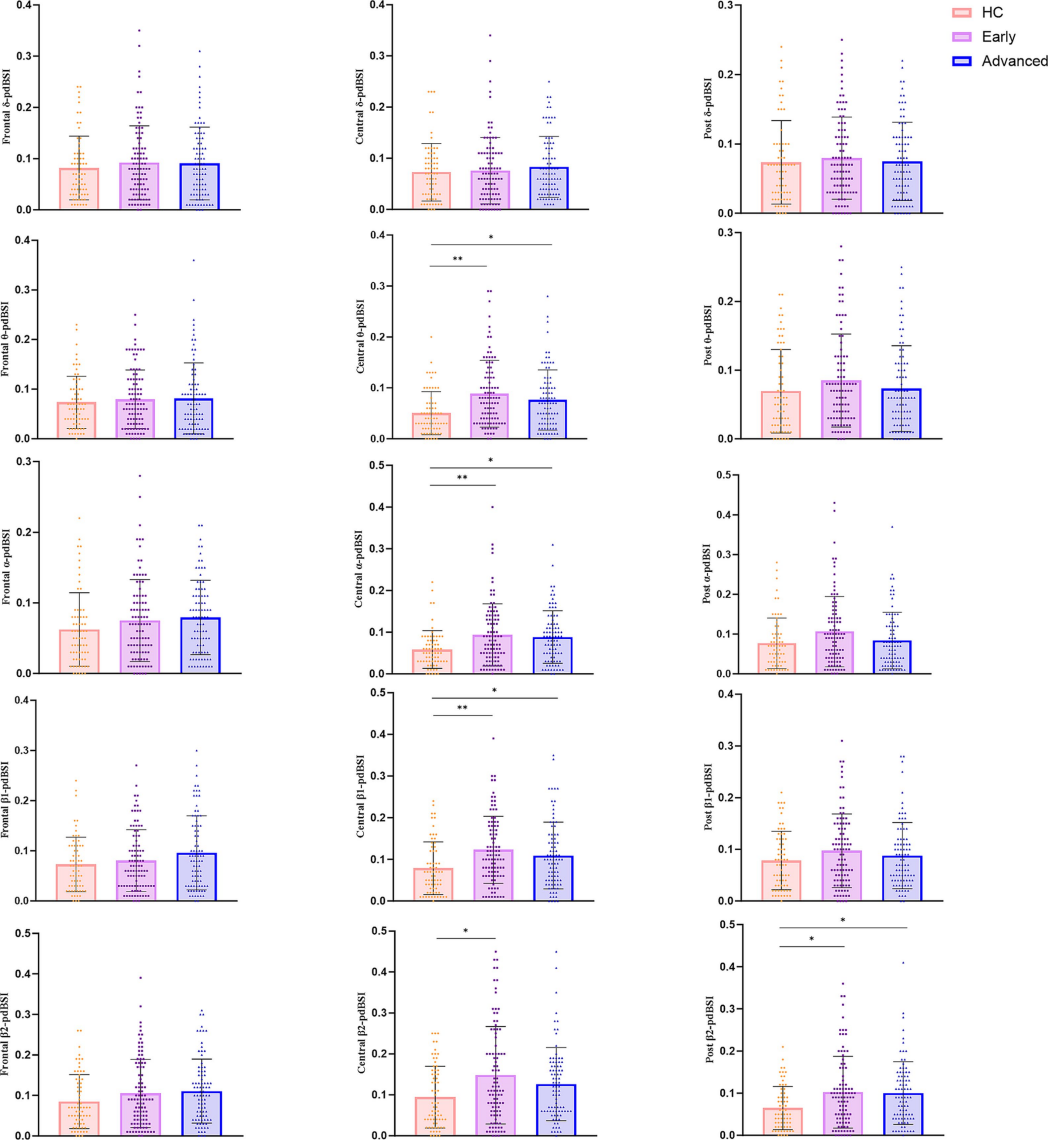
Differences in pdBSI across frequency bands in local brain regions. **(a–c)** *δ* band in the frontal, central and posterior regions. **(d–f)** *θ* band in the frontal, central and posterior regions. **(g–i)** *α* band in the frontal, central and posterior regions. **(j–l)** β1 band in the frontal, central and posterior regions. **(m–o)** β2 band in the frontal, central and posterior regions (^*^p < 0.05, ^**^p < 0.005).

We further conducted region- and frequency-band-specific analysis. The results demonstrated that the ePD group showed higher levels of *θ*-pdBSI (Figure 5e, *P* < 0.001), *α*-pdBSI (Figure 5h, *p* < 0.005), β1-pdBSI (Figure 5k, *P* < 0.001) and β2-pdBSI (Figure 5n, *P* < 0.05) in the central region than the HC group. The same tendency was observed between the aPD and HC group. The β2-pdBSI of the posterior regions in ePD and aPD were higher than the HC group (Figure 5o, *p* < 0.05). The results showed that ePD had the highest pdBSI level, followed by the aPD and HC group. No significant differences were found in the frontal region across all bands (Figures 5a, d, g, j, m), in the central region in the δ band (Figure 5b), and in the posterior region in the δ, θ, α, and β1 bands (Figures 5c, f, i, l).

### 3.3 Correlation between pdBSI and MDS-UPDRS scale

**Figure 6:**
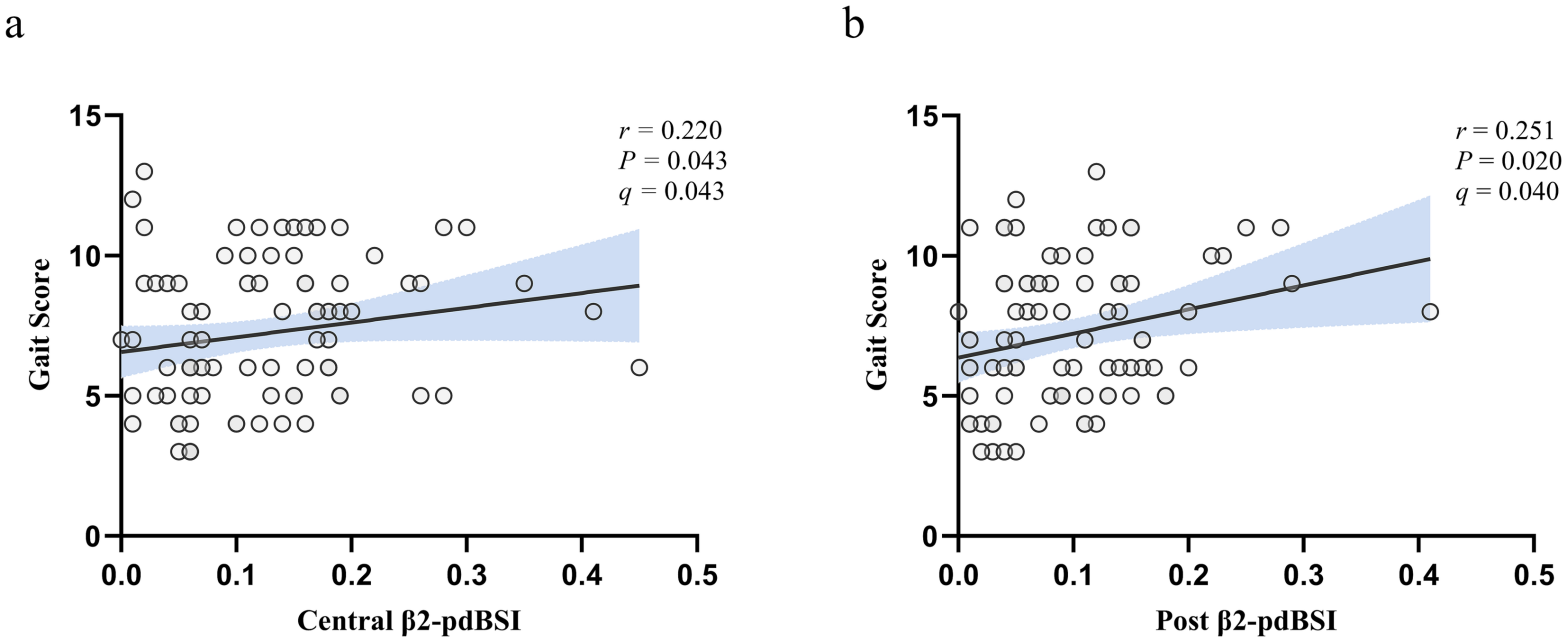
The correlation between pdBSI and UPDRS-III-gait score in aPD patients. **(a)** Correlation between β2-pdBSI in the central region and UPDRS-III gait score in aPD patients. **(b)** Correlation between β2-pdBSI in the posterior region and UPDRS-III gait score in aPD patients. (*r* denotes the correlation coefficient; *p* < 0.05 indicates significant correlation; *q* < 0.05 indicates significant correlation after FDR correction).

In order to clarify the association between pdBSI and UPDRS scale, our results showed that there was a positive correlation between β2-pdBSI of the central (*r* = 0.220, *p* = 0.043, *q* = 0.043, FDR corrected) and posterior regions (*r* = 0.251, *p* = 0.020, *q* = 0.040, FDR corrected) with the gait sub-score of the UPDRS-III in the aPD group. It indicates that interhemispheric symmetry in the β2 band is significantly associated with the severity of gait impairment (Figure 6).

### 3.4 Association between LEDD and pdBSI independent of disease stage

Having observed a trend-level difference in LEDD between PD stages (*p* = 0.054), we next examined whether medication dose independently predicted pdBSI. Partial correlation analyses, controlling for disease stage, revealed no significant associations between LEDD and pdBSI measures across the entire PD sample (all *p* > 0.05). This indicates that variance in dopaminergic medication dose was not a significant independent contributor to interhemispheric spectral asymmetry after accounting for disease stage (Supplementary Table 3).

## 4 Discussion

Early motor symptoms in PD are frequently asymmetric, typically manifesting unilaterally before progressing to bilateral involvement. This clinical lateralization is thought to mirror underlying asymmetric pathological changes, particularly the predominant degeneration of dopaminergic neurons in the contralateral substantia nigra. In this study, we applied the pdBSI—a quantitative metric of interhemispheric spectral power asymmetry—to investigate its relationship with disease stage and motor severity in PD. Our core finding is a distinct gradient of pdBSI values: ePD > aPD > HC. This pattern requires interpretation not merely as a measure of “more” or “less” abnormality, but as a dynamic electrophysiological signature of the disease’s pathological evolution.

The core pathology of PD involves the loss of dopaminergic neurons in the substantia nigra, which is markedly unilateral in the early stage. The asymmetry of motor symptoms in PD patients is associated with contralateral neurodegenerative changes and lateralized dopamine depletion at the level of the basal ganglia (BG) (Goedert et al., 2013; Barrett et al., 2011; Liu et al., 2024; Borghammer, 2021; Kaasinen, 2016; Kumakura et al., 2006). Dopamine Transporter Single Photon Emission Computed Tomography (DAT-SPECT) neuroimaging has revealed asymmetric impairment in both function and density of striatal dopamine transporters (DAT) in early-stage PD patients, whereas late-stage patients exhibit severe bilateral striatal dysfunction and reduced DAT density (Liu, 2007). The asymmetric cerebral pathological alterations lead to abnormal functional connectivity within motor networks, ultimately resulting in impaired motor coordination (Liuzzi et al., 2011). In our study, the H-Y stage was primarily based on motor symptom severity and postural stability, which was used to classify disease progression. The observed gradient (ePD > aPD > HC) offers a physiological interpretation of the clinical evolution from asymmetric to bilateral symptoms. In early-stage PD (ePD), pathological changes are markedly lateralized, leading to a strong interhemispheric contrast in oscillatory activity—captured as high pdBSI. As the disease advances to aPD, neurodegeneration and compensatory mechanisms likely involve the initially less-affected hemisphere, reducing the interhemispheric contrast. Thus, the decrease in pdBSI from ePD to aPD may not indicate “improvement” but rather a bilateralization of pathology, resulting in a more symmetrically abnormal oscillatory state across hemispheres. This interpretation aligns with the clinical observation of increasing bilateral motor deficits despite a potential reduction in the gross asymmetry metric.

Motor dysfunction in PD originates from disrupted neural activity across multiple interacting brain regions. In this study, the cerebral cortex was segmented into three functional regions, the anterior region (F7, F3, F4, F8), the central region (T3, C3, C4, T4), and the posterior region (T5, P3, P4, T6). Based on this parcellation, regional characteristics of the pdBSI were investigated. The results revealed that electroencephalographic spectral power asymmetry in PD patients was predominantly localized to the central and posterior regions. The M1 serves as a core structure in volitional motor control in humans. Midbrain dopaminergic neurons modulate neuronal firing rates and synchrony within M1 through both direct projections and indirect pathways involving the basal ganglia-thalamic circuitry (Galvan et al., 2015). The parietal cortex contributes to object-directed motor control and is crucial for postural stability, while the posterior parietal cortex integrates somatosensory information and plays a key role in lower limb motor control (Freund, 2003). Cortical thickness in specific brain areas decreases with increasing motor disability across PD stages. Early-stage PD patients often exhibit higher rates of cortical atrophy in the left insula and olfactory sulcus, whereas late-stage patients show more pronounced atrophy in bilateral occipital cortices (Claassen et al., 2016). The findings of this study demonstrate that alterations in pdBSI of PD patients are most prominent in the central and posterior regions. The concentration of pdBSI alterations in these areas underscores that hemispheric asymmetry in PD is not global but is functionally anchored to networks subserving motor planning, execution, and stability.

We further focused on the investigation of multi-band EEG activity. Existing evidence has demonstrated that cortical EEG activity in PD patients is generally slowed compared to healthy individuals. As early as the initial stages of the disease, fundamental EEG rhythms undergo alterations, characterized by a decrease in relative power in high-frequency bands and an increase in relative power in low-frequency bands (Wang et al., 2020). Neural oscillatory activity within the beta frequency range (13–35 Hz) is critically important for motor control, playing a role in movement regulation during both motor preparation and execution (van Wijk et al., 2012). Elevated beta activity in the motor cortex is associated with dopaminergic depletion in the brain (Mallet et al., 2008). Excessive beta-band oscillations have been recorded in the PD patients (Cassidy et al., 2002), and beta oscillations in the basal ganglia–subthalamic nucleus (STN) circuit are regarded as an electrophysiological hallmark of PD. Increased beta-band power has been empirically linked to several core motor symptoms of PD, including gait initiation failure, bradykinesia, and rigidity (Gatev et al., 2006; Oswal et al., 2013; Quynh et al., 2016).

Given the central role of the beta frequency band in the pathophysiology of PD, we further subdivided it into two functionally distinct sub-bands: low-beta (β1, 13–20 Hz) and high-beta (β2, 20–30 Hz). Synchronization in the β1 band is primarily involved in the regulation of movement execution and inhibition (Cao et al., 2024), while the functional mechanisms of the β2 band remain less clearly defined. Studies have shown that the intensity of β2 activity correlates positively with the severity of motor impairment in PD patients (Neumann et al., 2016), whereas improvement in motor symptoms is mainly associated with a reduction in β1 power (Priori et al., 2004). Moreover, PD patients with freezing of gait (FOG) often exhibit elevated β2 power, and a decrease in this band following levodopa administration is frequently accompanied by amelioration of FOG episodes (Toledo et al., 2014). The results of this study demonstrated a widespread elevation in both β1-pdBSI and β2-pdBSI across the whole brain in PD patients, with relative concentration in motor-related cortical regions. This suggests a broad dysregulation of beta-band symmetry and further supports the critical involvement of abnormal beta oscillations in PD motor dysfunction. These findings also indicate the presence of widespread asymmetric impairment in motor-related brain areas, leading to interhemispheric asymmetry in beta-band power. Furthermore, our results revealed that increased β2-pdBSI in the central and posterior region of the aPD group correlated positively with the UPDRS-III gait subscore. This suggests that more severe gait disturbances in aPD may be reflected by changes in β2-pdBSI, and that disrupted beta oscillatory symmetry in posterior sensory integration areas may serve as a key electrophysiological substrate for gait impairment. Specifically, the elevation of β2-pdBSI in posterior regions and its correlation with gait impairment in aPD suggest that even in advanced stages, a residual or evolving asymmetry in sensorimotor integration circuits is clinically significant. This asymmetry in the high-beta band may represent a maladaptive signature that persists despite broader bilateral pathology and higher medication doses.

Furthermore, this study identified significant differences in pdBSI within the theta and alpha frequency bands in PD patients, with the most prominent abnormalities localized to the central region. Additional evidence indicates that aberrant theta rhythm affects motor execution in PD patients with freezing of gait (Zampogna et al., 2022). Abnormal activities of the theta and alpha bands have been established as predictive indicators of cognitive impairment and the severity of motor symptoms in PD (Wang et al., 2020; Cozac et al., 2016; Palmer et al., 2010). Our observation of altered pdBSI in theta and alpha bands, primarily in the central region, further confirms the widespread dysregulation of oscillatory symmetry in PD. While these findings did not correlate with UPDRS scores in our cohort—potentially due to sample size or the predominant focus on motor items—they enrich the picture of multi-frequency network imbalance. The convergence of asymmetry across multiple bands underscores that PD affects interhemispheric balance broadly, not within a single oscillatory system.

## 5 Limitations and future directions

This study has several limitations. Firstly, all EEG and clinical assessments were conducted exclusively in the dopaminergic medication-ON state. While this design enhances clinical applicability and was statistically controlled for LEDD, it cannot separate disease-related neural asymmetry from medication-induced modulation of oscillatory activity. Future studies incorporating OFF-medication assessments or within-subject ON–OFF designs are essential to confirm whether pdBSI reflects trait (disease) or state (medication) characteristics. Secondly, due to the limited sample size, the statistical power was reduced, which may have prevented the identification of more significant indicators. Additionally, the lack of imaging data made it impossible to correlate differences in EEG signals with imaging characteristics. Future studies should expand the sample size and incorporate imaging data to conduct subgroup analyses of lesions in the left and right hemispheres. Furthermore, integrating complementary measures such as gait kinematics or inertial measurement unit (IMU) data with EEG could enable a more comprehensive, multimodal analysis of motor dysfunction in PD.

## 6 Conclusion

There was hemispheric asymmetry in the PD patients. The pdBSI was a potential electrophysiological biomarker for hemispheric asymmetry in the PD. β2-pdBSI in the central and posterior region was significantly associated with the severity of gait impairment in aPD patients. In addition, the stage-dependent gradient elucidates the dynamic shift from unilateral to bilateral network involvement. Our findings underscore that quantifying interhemispheric asymmetry, beyond examining power in isolated regions, provides unique insights into the pathophysiology and clinical progression of PD.

## Data Availability

The raw data supporting the conclusions of this article will be made available by the authors, without undue reservation.
