# Supplementary material for: Correlation between brain symmetry index and motor function in Parkinson’s disease: a cross-sectional study
Source: Front Neurosci. 2026 Feb 24;20:1777368. doi: 10.3389/fnins.2026.1777368 (PMC12974094; doi:10.3389/fnins.2026.1777368)
Supplement: Supplementary file 1 [file Table_1.docx]

**Supplementary 1**

To avoid formatting inconsistencies of equations caused by different versions of Word, the equations in the manuscript are temporarily represented as images (Fig 2). Below are the LaTeX code and its rendered output for the equation [1]:

\[BSI = \frac{1}{8} \left ( \frac{\text{Fp2} - \text{Fp1}}{\text{Fp1} + \text{Fp2}} + \frac{\text{F4} - \text{F3}}{\text{F3} + \text{F4}} + \frac{\text{C4} - \text{C3}}{\text{C3} + \text{C4}} + \frac{\text{P4} - \text{P3}}{\text{P3} + \text{P4}} + \frac{\text{O2} - \text{O1}}{\text{O1} + \text{O2}} + \frac{\text{F8} - \text{F7}}{\text{F7} + \text{F8}} + \frac{\text{T4} - \text{T3}}{\text{T3} + \text{T4}} + \frac{\text{T6} - \text{T5}}{\text{T5} + \text{T6}} \right)\]

$$BSI=\frac{1}{8}\left( \frac{Fp2-Fp1}{Fp1+Fp2}+\frac{F4-F3}{F3+F4}+\frac{C4-C3}{C3+C4}+\frac{P4-P3}{P3+P4}+\frac{O2-O1}{O1+O2}+\frac{F8-F7}{F7+F8}+\frac{T4-T3}{T3+T4}+\frac{T6-T5}{T5+T6} \right)$$

**Supplementary 2-table 1**

**Comparison of pdBSI between three groups of different brain regions**

| pdBSI | Brain regions | HC group | ePD group | aPD group | Z | *P* |
| --- | --- | --- | --- | --- | --- | --- |
| all-pdBSI | Global | 0.03 (0.01, 0.05) | 0.05 (0.02, 0.08) | 0.05 (0.03, 0.08) | 12.015 | **0.002*** |
|  | Frontal | 0.06 (0.03, 0.90) | 0.06 (0.40, 0.10) | 0.07 (0.03, 0.11) | 1.462 | 0.482 |
|  | Central | 0.05 (0.015, 0.08) | 0.09 (0.04, 0.135) | 0.07 (0.03, 0.012) | 21.631 | **<0.001*** |
|  | Posterior | 0.05 (0.02, 0.085) | 0.07 (0.03, 0.11) | 0.06 (0.04, 0.10) | 5.972 | 0.050 |
| δ-pdBSI | Global | 0.05 (0.02, 0.08) | 0.045 (0.02, 0.075) | 0.04 (0.02, 0.09) | 0.040 | 0.980 |
|  | Frontal | 0.07 (0.03, 0.11) | 0.08 (0.04, 0.12) | 0.08 (0.03, 0.13) | 0.576 | 0.750 |
|  | Central | 0.07 (0.03, 0.10) | 0.06 (0.03, 0.11) | 0.07 (0.04, 0.12) | 1.385 | 0.500 |
|  | Posterior | 0.05 (0.02, 0.085) | 0.07 (0.03, 0.115) | 0.06 (0.03, 0.11) | 0.651 | 0.722 |
| θ-pdBSI | Global | 0.03 (0.02, 0.07) | 0.045 (0.02, 0.085) | 0.05 (0.02, 0.07) | 1.263 | 0.532 |
|  | Frontal | 0.06 (0.04, 0.10) | 0.07 (0.03, 0.12) | 0.06 (0.02, 0.11) | 0.200 | 0.905 |
|  | Central | 0.04 (0.02, 0.07) | 0.075 (0.035, 0.125) | 0.07 (0.03, 0.10) | 16.690 | **<0.001*** |
|  | Posterior | 0.05 (0.02, 0.115) | 0.07 (0.03, 0.12) | 0.06 (0.03, 0.11) | 3.155 | 0.206 |
| α-pdBSI | Global | 0.03 (0.02, 0.055) | 0.06 (0.025, 0.10) | 0.05 (0.03, 0.09) | 14.420 | \| **<0.001*** \| \| --- \| |
|  | Frontal | 0.05 (0.25, 0.08) | 0.07 (0.03, 0.105) | 0.08 (0.03, 0.11) | 5.059 | 0.080 |
|  | Central | 0.05 (0.03, 0.08) | 0.075 (0.04, 0.135) | 0.08 (0.04, 0.12) | 12.236 | **0.002*** |
|  | Posterior | 0.07 (0.03, 0.10) | 0.09 (0.04, 0.140) | 0.07 (0.03, 0.12) | 5.264 | 0.072 |
| β1-pdBSI | Global | 0.04 (0.02, 0.06) | 0.07 (0.03, 0.10) | 0.07 (0.03, 0.09) | 10.683 | **0.005*** |
|  | Frontal | 0.07 (0.03, 0.11) | 0.065 (0.03, 0.115) | 0.08 (0.03, 0.150) | 2.905 | 0.234 |
|  | Central | 0.07 (0.03, 0.12) | 0.11 (0.06, 0.18) | 0.10 (0.05, 0.15) | 13.746 | **0.001*** |
|  | Posterior | 0.07 (0.03, 0.12) | 0.09 (0.04, 0.145) | 0.07 (0.04, 0.12) | 2.382 | 0.304 |
| β2p-dBSI | Global | 0.04 (0.02, 0.07) | 0.07 (0.025, 0.12) | 0.08 (0.03, 0.11) | 9.495 | **0.009*** |
|  | Frontal | 0.07 (0.03, 0.135) | 0.09 (0.035, 0.017) | 0.10 (0.04, 0.015) | 3.923 | 0.141 |
|  | Central | 0.07 (0.035, 0.115) | 0.11 (0.055, 0.21) | 0.12 (0.06, 0.17) | 8.454 | **0.015*** |
|  | Posterior | 0.05 (0.02, 0.095) | 0.09 (0.40, 0.135) | 0.09 (0.04, 0.14) | 10.549 | **0.005*** |

**Note.** Kruskal-Wallis test: **P<*0.05. ePD, patients with Parkinson's disease at the early stage; aPD, patients with Parkinson's disease at the advanced stage; HC, health controls; pdBSI, pair wise derived brain symmetry index. all-pdBSI, The pdBSI of full-frequency band.

**Supplementary 3-table 2**

**Correlation between pdBSI and MDS-UPDRS in ePD patients**

| pdBSI | Brain regions | UPDRS-I  (*r* / *P*) | UPDRS-II  (*r* / *P*) | UPDRS-III  (*r* / *P*) | UPDRS-total  (*r* / *P*) | Rigidity  (*r* / *P*) | Bradykinesia  (*r* / *P*) | Tremor  (*r* / *P*) | Gait  (*r* / *P*) |
| --- | --- | --- | --- | --- | --- | --- | --- | --- | --- |
| all-pdBSI | Global | 0.019/0.851 | 0.084/0.471 | 0.104/0.313 | 0.077/0.458 | 0.108/0.296 | 0.091/0.378 | -0.138/0.179 | 0.013/0.903 |
|  | Frontal | 0.046/0.653 | -0.039/0.707 | -0.171/0.095 | -0.132/0.201 | -0.168/0.101 | -0.147/0.153 | 0.006/0.957 | -0.136/0.186 |
|  | Central | -0.113/0.274 | 0.078/0.451 | 0.003/0.981 | -0.031/0.761 | 0.098/0.344 | -0.038/0.711 | -0.097/0.345 | -0.050/0.626 |
|  | Posterior | -0.05/0.626 | -0.061/0.553 | 0.077/0.458 | 0.009/0.932 | 0.084/0.416 | 0.054/0.599 | -0.028/0.789 | 0.020/0.847 |
| δ-pdBSI | Global | -0.101/0.33 | -0.105/0.306 | -0.233/0.023 | -0.214/0.036 | -0.208/0.042 | -0.185/0.070 | -0.025/0.812 | -0.297/0.003 |
|  | Frontal | -0.088/0.395 | 0.071/0.489 | -0.177/0.084 | -0.147/0.153 | -0.181/0.077 | -0.176/0.087 | 0.006/0.953 | -0.138/0.180 |
|  | Central | -0.096/0.353 | -0.128/0.231 | -0.171/0.096 | -0.150/0.145 | -0.137/0.182 | -0.177/0.084 | 0.070/0.498 | -0.270/0.008 |
|  | Posterior | -0.093/0.367 | -0.235/0.021 | -0.170/0.098 | -0.206/0.144 | -0.082/0.427 | -0.109/0.290 | -0.070/0.499 | -0.202/0.048 |
| θ-pdBSI | Global | 0.088/0.393 | -0.019/0.851 | 0.017/0.869 | 0.031/0.767 | 0.058/0.573 | 0.031/0.763 | -0.077/0.458 | 0.004/0.970 |
|  | Frontal | 0.106/0.303 | -0.044/0.671 | -0.127/0.218 | -0.060/0.562 | -0.123/0.231 | -0.175/0.088 | 0.050/0.630 | -0.021/0.837 |
|  | Central | -0.196/0.055 | -0.154/0.134 | -0.094/0.360 | -0.160/0.119 | -0.163/0.122 | 0.071/0.494 | -0.055/0.595 | -0.153/0.137 |
|  | Posterior | -0.102/0.323 | -0.093/0.366 | -0.083/0.421 | -0.088/0.394 | -0.022/0.832 | -0.125/0.223 | 0.014/0.892 | -0.045/0.661 |
| α-pdBSI | Global | 0.029/0.783 | 0.010/0.921 | -0.054/0.604 | -0.051/0.621 | -0.038/0.711 | 0.020/0.843 | -0.134/0.192 | -0.002/0.987 |
|  | Frontal | 0.077/0.455 | 0.065/0.515 | 0.002/0.998 | 0.032/0.757 | -0.095/0.359 | 0.005/0.963 | 0.036/0.726 | 0.054/0.601 |
|  | Central | 0.03/0.769 | 0.071/0.494 | -0.053/0.606 | -0.034/0.742 | -0.114/0.269 | -0.036/0.730 | -0.034/0.745 | -0.054/0.601 |
|  | Posterior | -0.09/0.384 | -0.096/0.350 | 0.010/0.927 | -0.043/0.675 | -0.061/0.554 | -0.033/0.748 | 0.105/0.310 | -0.097/0.349 |
| β1-pdBSI | Global | -0.031/0.768 | 0.090/0.384 | 0.104/0.314 | 0.074/0.472 | 0.089/0.387 | 0.068/0.509 | -0.125/0.224 | 0.117/0.257 |
|  | Frontal | 0.112/0.274 | -0.002/0.981 | -0.129/0.209 | -0.151/0.307 | -0.119/0.248 | -0.106/0.304 | -0.108/0.294 | 0.033/0.748 |
|  | Central | -0.031/0.762 | -0.012/0.908 | -0.081/0.430 | -0.095/0.359 | 0.105/0.307 | -0.096/0.350 | -0.143/0.166 | -0.066/0.520 |
|  | Posterior | -0.022/0.832 | -0.099/0.366 | 0.032/0.759 | -0.015/0.886 | 0.092/0.375 | -0.013/0.903 | -0.051/0.622 | 0.083/0.422 |
| β2-pdBSI | Global | -0.076/0.464 | 0.020/0.846 | 0.091/0.379 | 0.071/0.493 | 0.249/0.014 | 0.078/0.450 | -0.072/0.486 | -0.015/0.882 |
|  | Frontal | 0.069/0.504 | 0.059/0.565 | -0.126/0.222 | 0.074/0.473 | -0.108/0.295 | -0.110/0.287 | -0.039/0.704 | 0.077/0.458 |
|  | Central | -0.100/0.332 | 0.023/0.826 | -0.082/0.427 | -0.090/0.381 | 0.152/0.140 | -0.047/0.649 | -0.196/0.055 | -0.024/0.815 |
|  | Posterior | -0.107/0.302 | -0.056/0.586 | 0.151/0.143 | 0.087/0.401 | 0.274/0.015 | 0.097/0.384 | 0.073/0.477 | -0.059/0.570 |

**Correlation between pdBSI and MDS-UPDRS in aPD patients**

| pdBSI | Brain regions | UPDRS-I  (*r* / *P*) | UPDRS-II  (*r* / *P*) | UPDRS-III  (*r* / *P*) | UPDRS-all  (*r* / *P*) | Rigidity  (*r* / *P*) | Bradykinesia  (*r* / *P*) | Tremor  (*r* / *P*) | Gait  (*r* / *P*) |
| --- | --- | --- | --- | --- | --- | --- | --- | --- | --- |
| all-pdBSI | Global | 0.069/0.531 | 0.041/0.709 | -0.045/0.680 | -0.024/0.825 | -0.125/0.256 | -0.077/0.482 | 0.118/0.284 | -0.048/0.665 |
|  | Frontal | -0.004/0.972 | -0.023/0.833 | -0.213/0.051 | -0.161/0.141 | 0.071/0.521 | -0.203/0.062 | -0.039/0.726 | -0.221/0.042 |
|  | Central | 0.049/0.653 | 0.015/0.892 | 0.04/0.719 | 0.049/0.654 | -0.125/0.255 | -0.008/0.941 | 0.237/0.029 | 0.018/0.867 |
|  | Posterior | 0.011/0.920 | -0.058/0.596 | -0.036/0.742 | -0.049/0.655 | -0.139/0.206 | -0.066/0.548 | 0.034/0.759 | -0.020/0.855 |
| δ-pdBSI | Global | 0.025/0.819 | 0.01/0.924 | 0.010/0.930 | 0.010/0.926 | 0.065/0.554 | -0.042/0.704 | 0.128/0.244 | 0.088/0.426 |
|  | Frontal | -0.052/0.365 | 0.027/0.805 | 0.043/0.693 | 0.022/0.842 | -0.009/0.938 | 0.033/0.762 | 0.148/0.176 | -0.100/0.365 |
|  | Central | 0.010/0.926 | 0.010/0.931 | -0.066/0.550 | -0.024/0.824 | -0.035/0.752 | -0.104/0.345 | 0.221/0.042 | -0.045/0.682 |
|  | Posterior | -0.020/0.857 | -0.159/0.147 | -0.127/0.247 | -0.151/0.169 | -0.214/0.050 | -0.120/0.272 | 0.036/0.745 | -0.115/0.295 |
| θ-pdBSI | Global | 0.038/0.730 | 0.041/0.707 | 0.023/0.833 | 0.043/0.698 | 0.067/0.545 | -0.031/0.780 | 0.100/0.363 | -0.030/0.786 |
|  | Frontal | 0.145/0.187 | 0.145/0.184 | 0.042/0.699 | 0.115/0.294 | 0.311/0.004 | -0.014/0.896 | 0.115/0.294 | -0.055/0.617 |
|  | Central | 0.105/0.340 | 0.151/0.169 | 0.109/0.321 | 0.146/0.182 | 0.066/0.551 | 0.049/0.659 | 0.247/0.023 | 0.074/0.504 |
|  | Posterior | -0.121/0.269 | -0.182/0.095 | -0.161/0.141 | -0.18/0.099 | 0.009/0.938 | -0.212/0.051 | 0.008/0.944 | -0.076/0.491 |
| α-pdBSI | Global | 0.026/0.815 | -0.072/0.515 | -0.136/0.214 | -0.137/0.210 | -0.055/0.618 | -0.154/0.159 | 0.096/0.380 | -0.171/0.117 |
|  | Frontal | 0.073/0.507 | 0.011/0.920 | -0.062/0.573 | -0.043/0.693 | 0.062/0.575 | -0.117/0.285 | 0.041/0.708 | -0.132/0.230 |
|  | Central | 0.115/0.294 | 0.022/0.844 | 0.018/0.868 | 0.025/0.820 | 0.002/0.985 | -0.012/0.911 | 0.148/0.176 | -0.079/0.470 |
|  | Posterior | 0.010/0.924 | -0.114/0.299 | -0.050/0.650 | -0.061/0.578 | -0.080/0.466 | -0.027/0.809 | 0.005/0.961 | 0.034/0.758 |
| β1-pdBSI | Global | 0.090/0.411 | 0.099/0.369 | 0.02/0.854 | 0.040/0.715 | -0.116/0.292 | 0.018/0.870 | 0.144/0.190 | -0.023/0.835 |
|  | Frontal | -0.024/0.826 | 0.024/0.829 | -0.177/0.105 | -0.132/0.228 | -0.093/0.398 | -0.167/0.125 | 0.008/0.939 | -0.123/0.261 |
|  | Central | 0.072/0.514 | 0.022/0.842 | 0.092/0.404 | 0.092/0.403 | -0.121/0.269 | 0.004/0.973 | 0.284/0.008* | 0.063/0.564 |
|  | Posterior | 0.038/0.728 | 0.013/0.906 | 0.102/0.351 | 0.058/0.595 | -0.012/0.915 | 0.048/0.666 | 0.135/0.217 | 0.040/0.717 |
| β2-pdBSI | Global | 0.022/0.841 | 0.104/0.344 | 0.047/0.667 | 0.071/0.521 | 0.001/0.99 | -0.028/0.801 | 0.043/0.696 | 0.153/0.161 |
|  | Frontal | -0.019/0.863 | 0.081/0.459 | -0.035/0.748 | -0.014/0.896 | 0.011/0.918 | -0.043/0.697 | 0.022/0.838 | -0.041/0.711 |
|  | Central | -0.077/0.481 | 0.004/0.969 | 0.093/0.397 | 0.074/0.500 | -0.097/0.376 | 0.036/0.746 | 0.074/0.501 | **0.220/0.043*** |
|  | Posterior | 0.087/0.427 | 0.171/0.119 | 0.093/0.398 | 0.152/0.165 | -0.039/0.723 | -0.02/0.853 | 0.042/0.702 | **0.251/0.020*** |

**Note.** Spearman correlation: *r* represents the correlation coefficient; *r* > 0 indicates a positive correlation, while *r* < 0 indicates a negative correlation.**P*＜0.05. ePD, patients with parkinson’s disease at the early stage;aPD, patients with parkinson’s disease at the advanced stage; MDS-UPDRS, the Movement Disorder Society-Sponsored Revision of the Unified Parkinson's Disease Rating Scale; MDS-UPDRS I-III, the part I-III of MDS-UPDRS; Rigidity, Bradykinesia, Tremor, Gait: the subitems of UPDRS III, rigidity (item 3.3), bradykinesia (items 3.4~3.8, 3.14), tremor (3.15~3.18), and gait (3.9~3.13). pdBSI-all,The pdBSI of full-frequency band. The β2-pdBSI in the central and posterior regions showed a significant correlation with gait scores, with q-value of 0.043(central β2-pdBSI) and 0.040 (posterior β2-pdBSI) after FDR correction.

**Supplementary 4-table3**

**Association between LEDD and pdBSI independent of disease stage**

| pdBSI | Brain regions | LEDD  (*r* / *P*) |
| --- | --- | --- |
| all-pdBSI | Global | 0.106/0.157 |
|  | Frontal | 0.65/0.381 |
|  | Central | \| 0.064/0.391 \| \| --- \| |
|  | Posterior | 0.014/0.854 |
| δ-pdBSI | Global | 0.066/0.376 |
|  | Frontal | 0.013/0.860 |
|  | Central | 0.063/0.401 |
|  | Posterior | -0.076/0.309 |
| θ-pdBSI | Global | 0.076/0.308 |
|  | Frontal | -0.009/0.907 |
|  | Central | 0.025/0.735 |
|  | Posterior | -0.004/0.962 |
| α-pdBSI | Global | 0.040/0.592 |
|  | Frontal | 0.000/0.315 |
|  | Central | 0.103/0.166 |
|  | Posterior | -0.075/0.315 |
| β1-pdBSI | Global | 0.080/0.282 |
|  | Frontal | 0.088/0.237 |
|  | Central | 0.060/0.421 |
|  | Posterior | -0.028/0.711 |
| β2-pdBSI | Global | 0.029/0.696 |
|  | Frontal | 0.064/0.388 |
|  | Central | 0.031/0.679 |
|  | Posterior | -0.001/0.992 |

**Note.**Partial correlations (controlling for disease stage) were calculated between LEDD and pdBSI in the overall PD sample. No statistically significant associations were found (all *P* > 0.05), indicating that dopaminergic medication dose was not independently linked to interhemispheric asymmetry after accounting for disease severity.

**References**

[1] Schleiger E, Sheikh N, Rowland T, Wong A, Read S, Finnigan S. Frontal eeg delta/alpha ratio and screening for post-stroke cognitive deficits: the power of four electrodes. Int J Psychophysiol 2014;94(1):19-24.
